# Demonstration of a Label-Free and Low-Cost Optical Cavity-Based Biosensor Using Streptavidin and C-Reactive Protein

**DOI:** 10.3390/bios11010004

**Published:** 2020-12-24

**Authors:** Donggee Rho, Seunghyun Kim

**Affiliations:** Electrical and Computer Engineering Department, Baylor University, One Bear Place #97356, Waco, TX 76798, USA; donggee_rho@baylor.edu

**Keywords:** biosensors, optical cavity-based biosensor, biomarker detection

## Abstract

An optical cavity-based biosensor (OCB) has been developed for point-of-care (POC) applications. This label-free biosensor employs low-cost components and simple fabrication processes to lower the overall cost while achieving high sensitivity using a differential detection method. To experimentally demonstrate its limit of detection (LOD), we conducted biosensing experiments with streptavidin and C-reactive protein (CRP). The optical cavity structure was optimized further for better sensitivity and easier fluid control. We utilized the polymer swelling property to fine-tune the optical cavity width, which significantly improved the success rate to produce measurable samples. Four different concentrations of streptavidin were tested in triplicate, and the LOD of the OCB was determined to be 1.35 nM. The OCB also successfully detected three different concentrations of human CRP using biotinylated CRP antibody. The LOD for CRP detection was 377 pM. All measurements were done using a small sample volume of 15 µL within 30 min. By reducing the sensing area, improving the functionalization and passivation processes, and increasing the sample volume, the LOD of the OCB are estimated to be reduced further to the femto-molar range. Overall, the demonstrated capability of the OCB in the present work shows great potential to be used as a promising POC biosensor.

## 1. Introduction

The early diagnosis of diseases, including cancers, infectious diseases, and cardiovascular diseases, is vital in order to apply effective treatments and increase the chance of full recovery [1,2,3,4]. Diagnostic technologies in the current healthcare system are mostly used at centralized laboratories, involve costly and time-consuming processes, and are operated by expert staff [2,5,6]. For example, enzyme-linked immunosorbent assay (ELISA), considered as the gold standard diagnostic method, is labor-intensive, requiring complicated procedures such as labeling and multiple washing steps [2,5,7,8]. Label-free optical biosensing methods such as surface plasmon resonance (SPR) and total internal reflection ellipsometry (TIRE) have been extensively investigated and developed [9,10]. SPR and TIRE biosensors are label-free biosensors without complex procedures, and are highly sensitive with reduced assay times. However, some drawbacks, including high-cost, bulky size, and the need for trained personnel to operate, remain to be improved [11,12]. With these limitations in the current diagnostic technologies, it is difficult for people to monitor their health status regularly, which would eventually increase the chance of being diagnosed with diseases at late stages [3,7]. The problems become worse for people who are in financial difficulties and living in developing countries with deficient healthcare systems [6,13]. To address these challenges existing in the conventional diagnostic field, a point-of-care (POC) biosensor has emerged as a promising alternative, allowing patients to regularly check their health condition at the bedside or near them without being dependent on central laboratory testing [4,6,14,15,16,17]. According to the World Health Organization, an ideal POC test should satisfy the ASSURED (affordable, sensitive, specific, user-friendly, rapid, equipment-free, deliverable to end-users) criteria [16,17]. One of the most widely available and commercialized POC devices is based on lateral flow assays (LFAs) with their low cost, ease of use, and speed [4,6,18]. However, limitations still remain with regard to LFAs in terms of producing reproducible and sensitive test results [6,18,19].

An optical cavity-based biosensor (OCB) using a differential detection method has been developed for the application of POC diagnostics [20,21,22,23,24,25,26,27]. The structure of an optical cavity consists of two partially reflective surfaces in parallel, separated by a small gap in between. The light propagating through the optical cavity experiences multiple beam interference due to those two reflective surfaces, and produces a transmission spectrum with a resonant characteristic. Because of the resonant characteristic, the optical cavity can be used to detect small changes inside the cavity which, in turn, makes it an attractive platform for biosensing applications. To use the optical cavity for biosensing, the sensing area is functionalized with receptor molecules. When target biomolecules are adsorbed into the receptors, a shift in the resonant response occurs. To detect the small resonant response shift, the OCB measures the changes in optical intensities at two different wavelengths using low-cost laser diodes and a CMOS camera instead of using an expensive spectrometer or a tunable laser, lowering the total cost. The sensitivity of the OCB is enhanced by employing a differential detection method. We designed the optical cavity structure so that the optical intensities of two wavelengths are changing in opposite directions upon the capture of target biomolecules, in order to have a significantly bigger change from the calculated differential values. The differential detection method not only increases the sensitivity but also offers other benefits for biosensing, such as power equalization (no initial power variation depending on the measurement results), a larger dynamic range (the detectable concentration range of the biomolecules), and a larger fabrication tolerance [23,28,29]. The intensity measurement method also enables the simultaneous detection of multiple analytes by immobilizing corresponding bioreceptors at different areas of the optical cavity surface where the laser beams pass through. The capability of this OCB to detect small changes in bulk refractive index was demonstrated by using refractive index fluids with proven portability [25,27]. As a preliminary test to confirm the application of OCB in detecting the binding events at the optical cavity sensing area, the attachment of biotinylated bovine serum albumin (BSA) was measured on the streptavidin-functionalized surface [26].

In this present work, we demonstrate the OCB with streptavidin and C-reactive protein (CRP), and determine the limit of detection (LOD) for these. The optimized optical cavity design with simulations, surface functionalization steps, testing procedures, and measurement results are discussed. We report the use of the OCB for biomarker (CRP) detection for the first time.

## 2. Materials and Methods

### 2.1. Materials

(3-Aminopropyl) triethoxysilane (99%, APTES), streptavidin (lyophilized solid), and bovine serum albumin (lyophilized powder, BSA) were purchased from Sigma Aldrich, Inc, St. Louis, USA. Sulfo-NHS-Biotin (EZ-Link, powder) was purchased from Thermo Scientific, Inc, Waltham, USA. Tris-HCl buffer (1M, pH 8.0) was purchased from Bio Basic, Inc, Amherst, USA. Biotin-conjugated C-reactive protein antibody was purchased from Novus Biologicals, LLC, Centennial, USA. Human C-reactive protein (≥97%, CRP) was purchased from R&D Systems, Inc, Minneapolis, USA. Spin-on-glass (IC1-200, SOG) was purchased from Futurrex, Inc, Franklin, USA. SU8 photoresist (SU8-2005) was purchased from Kayaku Advanced Materials, Inc, Westborough, USA. UV glue (NOA 86H) was purchased from Norland Products, Inc, East Windsor, USA.

### 2.2. Schematic

**Figure 1:**
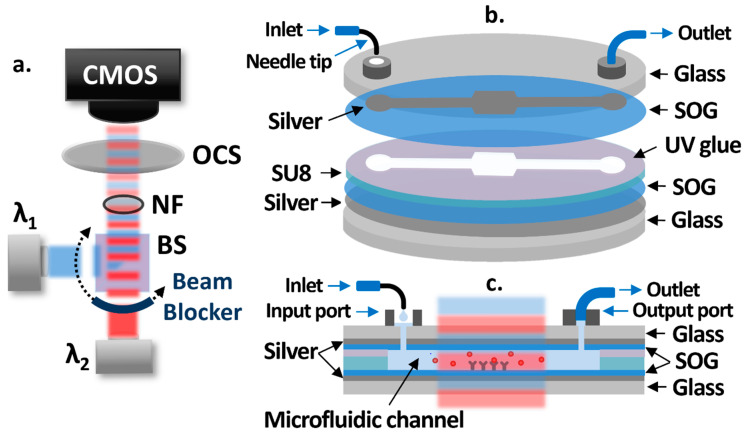
(**a**) Schematic diagram of the optical cavity-based biosensor (OCB) showing two laser beams at two different wavelengths (λ_1_ (**blue**) and λ_2_ (**red**)) alternatively propagating through the OCS with an interval of one second and reaching the CMOS camera. (**b**) Structure of the OCS showing each layer and connected input and output ports. (**c**) Cross-sectional view of the OCS showing the target biomolecule in a sample fluid being introduced into the microfluidic channel and attached to the receptor molecules on the SOG surface.

A schematic of the OCB is shown in Figure 1a. Two low-cost laser diodes at different wavelengths are used as light sources operated by laser diode drivers with the constant current mode. The laser beams are collimated, combined by a 50:50 beam splitter (BS), and alternatively propagate with one-second intervals using a rotating beam blocker. A neutral filter (NF) is placed in the light path to attenuate the intensities of laser beams in order to avoid the saturation of a CMOS camera (Discovery M15, Tucsen). The intensities of laser beams, transmitted through an optical cavity sample (OCS), are measured by the CMOS camera in real-time. Figure 1b shows each layer of the OCS structure, while Figure 1c shows the cross section of it. The bottom and patterned top silver layers on 3-inch glass substrates act as partially reflective surfaces. Spin-on-glass (SOG) layers are spin-coated on top of the silver layers to protect them from possible damages during the sample fabrication process and test, to facilitate the silanization-based surface functionalization process, and to maximize the sensitivity. The microfluidic channel between SOG layers is created by SU8 patterns. The receptor molecules are functionalized at the center area of the microfluidic channel, creating a sensing area. UV glue is used to bond the two separately processed substrates at the end of the fabrication process. To introduce fluids to the OCS without air bubbles, a syringe pump is used to add drops of fluids into the 3D printed input port (volume capacity: 20 μL) through a bent syringe tip, while a low-cost vacuum pump is attached to the 3D printed output port through tygon tubing to pull fluids from the input port through the microfluidic channel.

### 2.3. Simulation Results

As illustrated in Figure 1c, the target biomolecules in the sample fluid attach to the receptor molecules which, in turn, causes output intensity changes in the two laser diodes. For simulations, we employed the fixed index model enabling the approximation of the number of the biomolecules attached to receptors on the sensing area to a sensing layer thickness with a fixed refractive index [30]. We set the refractive index of the sensing layer to 1.45, which has been widely accepted for various biomolecules such as proteins, DNAs, and viruses [30,31,32,33]. FIMMWAVE/FIMMPROP (Photon design) was used to perform the simulations. For benefits such as enhanced sensitivity, power equalization, a larger dynamic range, and a larger fabrication tolerance, we employed a differential detection method [23,25]. For the differential detection method, a differential value (η) is calculated by the equation below.

$$\eta =\frac{I_{1}-I_{10}}{I_{10}}-\frac{I_{2}-I_{20}}{I_{20}}$$

*I*_1_ and *I*_2_ are the intensities (efficiencies for simulations) of λ_1_ and λ_2_, respectively, and *I*_10_ and *I*_20_ are the initial intensities for *I*_1_ and *I*_2_, respectively [26].

To achieve the largest differential value change, we searched for the optimal cavity width at which the efficiencies of two different wavelengths (out of available low-cost laser diodes in the market) change the most in the opposite directions with the sensing layer thickness increase. Since many different possible solutions exist, we narrowed our search to use a silver thickness of 20 nm with the microfluidic channel height ranging between 5 µm and 10 µm. We chose this channel height range to limit the fluid volume required to fill the channel without significant fluid flow resistivity. Since the fluid flow resistivity is inversely proportional to the third power of the height, if the height is too small, then the flow rate is slower, and a stronger vacuum pump is necessary to handle the fluids. Considering that the typical size of proteins is less than 20 nm, we focused on the simulation for a sensing layer thickness up to 20 nm. From the simulation results, we found that the differential value change depends on the SOG thickness. This means the local refractive index change due to the sensing layer change is more influential on the resonant characteristic at certain locations inside the cavity, which must be related to the electromagnetic field distribution in the cavity. Based on the spin curve of the SOG, we considered the SOG thickness in the range of 150 nm to 450 nm.

**Figure 2:**
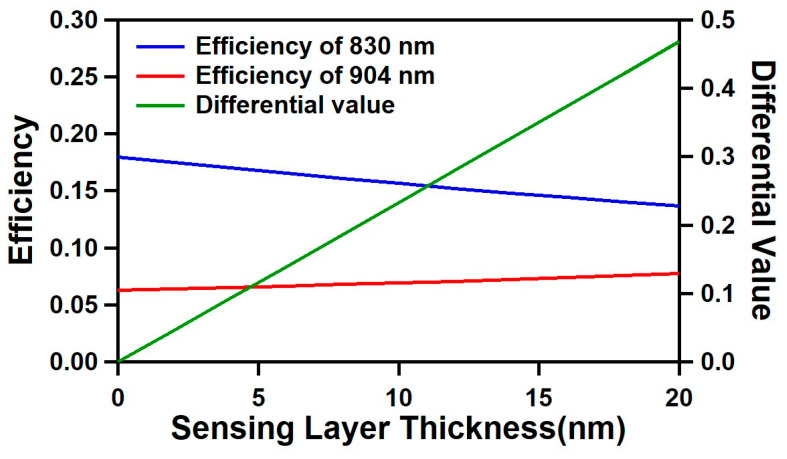
Simulation results showing efficiencies of 830 nm (**blue**) and 904 nm (**red**) wavelengths and differential values (**green**) versus the sensing layer thickness in the range between 0 and 20 nm.

The simulation results for the optimized optical cavity structure are shown in Figure 2. For the wavelengths of 830 nm (λ_1_) and 904 nm (λ_2_), the optimized optical cavity design has a cavity width (silver-to-silver distance) of 8 μm, and an SOG thickness of 400 nm with a silver thickness of 20 nm. As the sensing layer increased from 0 to 20 nm, the efficiency of 830 nm decreased from 0.18 to 0.137 (−0.043), while the efficiency of 904 nm increased from 0.063 to 0.077 (+0.014). With this opposite changing trend of two wavelengths, the corresponding differential value changed from 0 to 0.481, showing a significantly larger change compared to the individual wavelengths, with a better linearity.

### 2.4. Sample Fabrication and Surface Functionalization Processes

**Figure 3:**
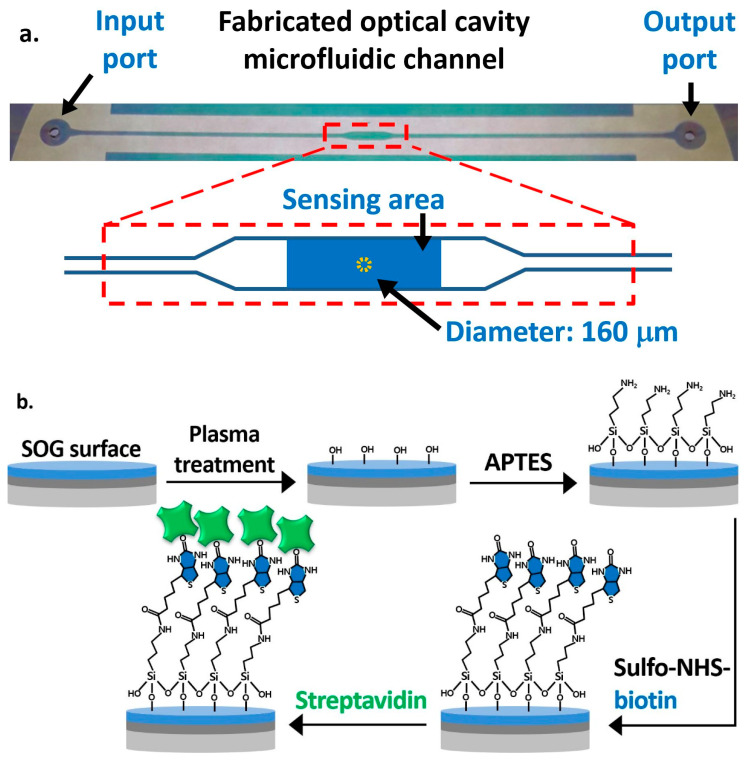
(**a**) Top view of the fabricated optical cavity microfluidic channel indicating the sensing area at the center and the area for the data process. (**b**) The functionalization procedure of the spin-on-glass (SOG) surface for the immobilization of streptavidin.

The fabrication process of the OCS is straightforward without complex micro and nano fabrication steps. First, a 3-inch glass substrate was drilled using a 1 mm diamond drill bit to make the inlet and outlet of a microfluidic channel. A silver layer was sputter-deposited on the drilled glass substrate and on another plain glass substrate. The top silver layer was patterned through a photolithography process followed by a wet-etch process, as shown in Figure 1b, for allowing UV illumination on the UV epoxy to cure and bond two substrates. Then, SOG was spin-coated at 1200 RPM on the silver layer of both substrates and cured at 130 °C on a hot plate. On top of the SOG layer of the plain glass substrate, an SU8 layer was patterned using a photolithography process to define the microfluidic channel. Finally, we used a UV curable epoxy to bond the drilled and plain glass substrates in order to form an optical cavity structure [26]. A top-view image of the fabricated optical cavity microfluidic channel is shown in Figure 3a. The typical layer thicknesses of fabricated devices are, on average, 22 nm (silver), 410 nm (SOG), 6.4 μm (SU8), and 1.08 μm (UV glue). The microfluidic channel has a total length of 5 cm, a height of 7.5 µm (distance between SOG surfaces), and a width of 500 µm, while the width at the sensing area is 1 mm. The sensing area is 2.5 mm^2^, and the dotted circular area at the center with a diameter of 160 μm represents the area used for the data processing, calculating the average intensities and differential values.

Figure 3b illustrates the functionalization steps on the SOG surface on the drilled substrate. The oxygen plasma treatment was applied for 5 min to create hydroxyl groups on the SOG surface. We performed the vapor-phase deposition of APTES by placing a substrate in a desiccator with 0.5 mL of 99% APTES in a small container placed at the bottom [34]. The entire desiccator was placed on a hot plate at 90 °C for 24 h to create terminal amine groups (-NH_2_) on the surface. After the overnight incubation, unbound APTES molecules were removed with DI water in an ultrasonic bath for 7 min, and the glass substrate was baked at 110 °C for 10 min. To functionalize the sensing area, 5 mg/mL of sulfo-NHS-biotin mixed in DI water was applied using a micropipette. It was then incubated for 1 h to covalently immobilize the biotin on the surface through amide bonds, while other areas were passivated with 1% BSA. The surface was then ready for the streptavidin detection experiment. BSA was also applied to the plain substrate with the SU8 pattern to passivate the bottom and side walls of the channel, so as to minimize the nonspecific binding of streptavidin and other biomolecules.

### 2.5. Test Setup

**Figure 4:**
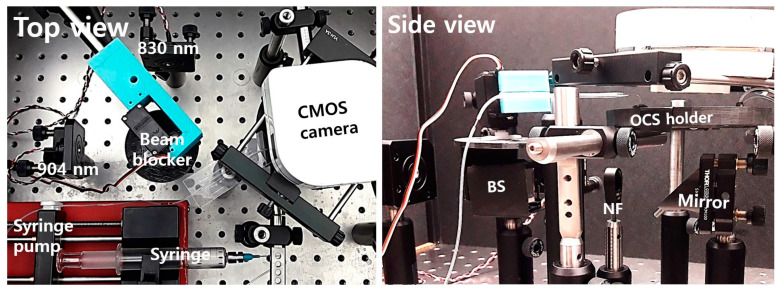
Top and side views of the test setup for the optical cavity-based biosensor (OCB).

Figure 4 shows the test setup on an optics table for experiments. Two laser diodes at 830 nm and 904 nm wavelengths were attached to collimators and mounted with kinematic mounts. A 3D-printed beam blocker with a servo motor was located on top of a 50:50 beam splitter to block the laser diode beams alternately. The side view in Figure 4 shows a BS, an NF, a 45-degree mirror under the fabricated OCS in a 3D-printed sample holder, and a CMOS camera. The total cost to build the whole system was low, at about USD 1100 excluding posts, mounts, and the syringe pump.

### 2.6. Fine-Tuning of the Optical Cavity Width Using Polymer Swelling

The optical response of any type of optical resonator is very sensitive to its cavity or resonator size. Due to possible errors during the fabrication process, the cavity widths of the fabricated OCSs show some variations. Even with a larger fabrication tolerance using the differential detection method, it is challenging to successfully fabricate the OCSs with a width accurate to within 40 nm, which leads to a low success rate in producing measurable samples [25]. We overcame this problem using the polymer swelling property [35,36,37]. The microfluidic channel walls were formed of SU8 and UV epoxy polymers. When the microfluidic channel was filled with DI water, the SU8 and UV epoxy in contact with DI water slowly swelled over time. As the optical cavity size increased due to the swelling, the optical intensities changed over time, following the resonance curve. At the optimal cavity width, it was anticipated that the intensities of the two wavelengths would change in opposite directions. During the swelling period, we monitored the optical intensity changes of both wavelengths and conducted sensing experiments when the optical cavity reached this optimal condition. The time it takes for this fine-tuning process varies from less than 1 h to more than 10 h, as the initial optical cavity widths differ. The swelling rate is rapid at the beginning and then slows over time. With this fine-tuning process, we were able to achieve a very high success rate of producing measurable samples (>90%).

## 3. Results

### 3.1. Streptavidin Detection

**Figure 5:**
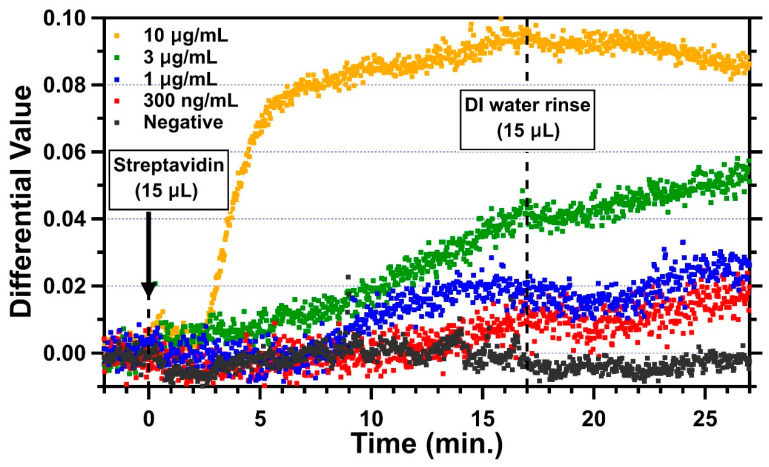
Real-time measurements for 30 min showing the changing differential values after the introduction of 15 μL of streptavidin for four different concentrations and the negative control (10 μg/mL: yellow; 3 μg/mL: green; 1 μg/mL: blue; 300 ng/mL: red; and negative control: black).

Streptavidin is a 52.8 kDa protein with a dimension of 5.6 nm × 4.2 nm × 4.2 nm [38]. For a monolayer of streptavidin with a height of 5.6 nm, the simulated differential value change due to this monolayer is 0.1266. For measurements, DI water was introduced first through the microfluidic channel for optical cavity width fine-tuning. When the optical cavity reached the measurable condition, 15 µL of streptavidin was then introduced with a flow rate of about 0.9 µL/min for about 17 min. Finally, the channel was rinsed with 15 µL of DI water. Representative trends of four different concentrations of streptavidin in DI water, 300 ng/mL, 1 µg/mL, 3 µg/mL, and 10 µg/mL, are shown in Figure 5 along with the negative control. The average differential value for 2 min before the introduction of streptavidin was set to 0 as the baseline. The change in the differential value due to the binding of streptavidin was measured by averaging differential values between 25 and 27 min, which is 8–10 min after the DI water was introduced for rinsing. For the negative control (black), the sensing area of this channel was blocked with BSA everywhere without sulfo-NHS-biotin. As expected, when 1 μg/mL of streptavidin was introduced into the negative control channel, no obvious change in the differential value was found, while there were some fluctuations during the period with streptavidin in between 0 and 17 min. This could have been due to the non-specific interaction of streptavidin with BSA. Clearly, some loosely attached streptavidin molecules were removed in the DI water rinse, and the differential values stabilized. The differential value change for the negative control was −0.00213, and the standard deviation was 0.00155. For the 10 µg/mL concentration (yellow), the differential value started changing in 2 min and reached 0.074 in 5.5 min with a slope of 0.0235/min after the introduction of streptavidin (at *t* = 0). The change slowed down from 5.5 min but kept increasing up to 0.095 with a slope of 0.00163/min until the DI water was introduced (at *t* = 17 min) for the rinse. As soon as the channel was rinsed, the change decreased slightly and reached 0.085 (at *t* = 27 min) on average. The result for the concentration of 3 µg/mL (green) shows that the differential value started slowly increasing at around 5 min and reached to 0.04 with a slope of 0.0023/min. The change stopped for about 3 min after the DI water rinse and then increased again to 0.055 with a slower slope of 0.0014/min. This could have been due to the binding of residual streptavidin molecules on the surface during the DI water rinse. The changes in differential values for the streptavidin concentrations of 1 µg/mL and 300 ng/mL started at 8 min and 12 min, respectively, with slower slopes (1 µg/mL: 0.00176/min; 300 ng/mL: 0.00089/min). After the introduction of DI water, they showed a similar trend whereby the differential value decreased for about 3 min and increased for the rest of the 7 min, reaching to 0.027 and 0.16, respectively. Those changes after the DI water rinse could also have been caused by the unbound molecules.

**Figure 6:**
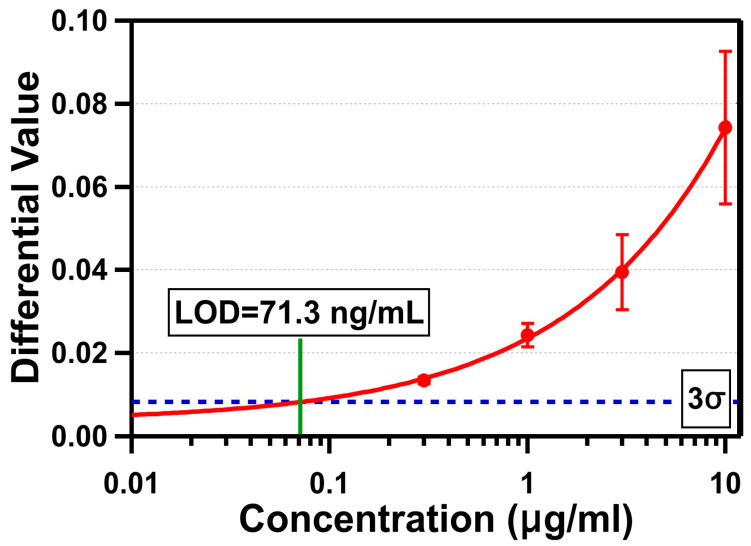
Differential values measured in triplicate versus four concentrations of streptavidin in a log scale.

The triplicate test results of four different concentrations are shown in Figure 6. The differential value due to the binding of streptavidin was measured by averaging differential values between 25 and 27 min, as described earlier. The average standard deviation of DI water was measured to be 0.00274. The average differential value changes were 0.074 ± 0.018 (10 µg/mL), 0.039 ± 0.0091 (3 µg/mL), 0.024 ± 0.003 (1 µg/mL), and 0.013 ± 0.001 (300 ng/mL). The LOD of our OCB biosensor was determined by the average sensor response crossing the 3σ line (0.00821), which was 71.3 ng/mL (1.35 nM). The differential value of 10 µg/mL did not reach the anticipated value for a monolayer of streptavidin (0.1266). There are a few possible hypotheses to explain this: (1) the streptavidin molecules on the sensing area were oriented towards where the smaller side of the molecule was in the beam propagation direction; (2) the actual refractive index change due to the immobilization of the streptavidin was less than the monolayer with the refractive index of 1.45 used for simulation; or (3) the functionalization and passivation processes were not sufficient to allow streptavidin molecules to form a densely-packed monolayer on the sensing area without losing them through non-specific bindings on other passivated surfaces. Out of these, the third is most likely. Even if the layer created by the immobilized streptavidin molecules was thinner with a lower refractive index, and the differential value for a monolayer of streptavidin was about 0.074 (average differential value change for 10 µg/mL), it is clear we were not able to form a densely packed streptavidin only on the sensing area. Based on the given size of the streptavidin, the total amount of streptavidin required to form a monolayer covering the entire sensing area of 2.5 mm^2^ is estimated to be 12.4 ng. For the streptavidin concentration of 1 μg/mL, the total amount of streptavidin in 15 µL of sample fluid is 15 ng. This indicates that, if all available streptavidin molecules are attached densely only on the sensing area, then there are more molecules than are necessary to form a monolayer. If a monolayer is formed and the assumption of the differential value change (0.074) for a monolayer of streptavidin is valid, then the differential value is supposed to reach that level with 1 μg/mL. However, since the differential value change for 1 μg/mL of concentration was only 0.024, the result clearly shows no monolayer was formed. This suggests the sensing area was not well functionalized with active biotin, and/or we lost many streptavidin molecules in other areas. If we improve the functionalization and passivation processes to block other areas of target biomolecules from being attached, so that all available target molecules are attached densely only on the sensing area, the result can be significantly improved.

### 3.2. C-Reactive Protein (CRP) Detection

**Figure 7:**
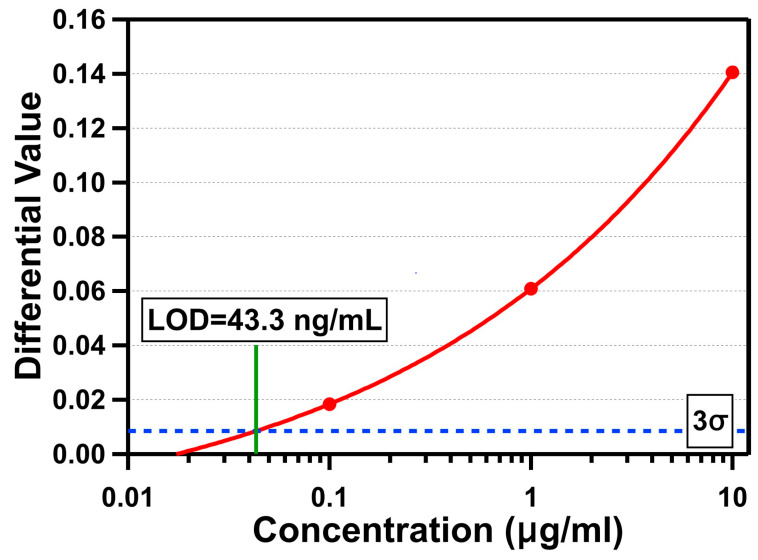
Differential values versus three concentrations of C-reactive protein (CRP) in a log scale.

CRP is a 115 kDa serum protein with a hydrated volume of 197.3 mm^3^, and is one of the most frequently used cardiac biomarkers with high specificity to diagnose and monitor cardiovascular diseases (CVDs), which are the leading cause of death worldwide [39]. The American Heart Association (AHA) and the Center for Diseases Control and Prevention (CDC) defined the risks of CVDs to be low for a concentration of CRP in humans below 1 μg/mL, moderate for a CRP concentration between 1 and 3 μg/mL, and high for a CRP concentration over 3 μg/mL [40]. The level of human CRP is also increased 1000-fold within 24–48 h in response to infection, inflammation, and tissue damage [41]. Figure 7 shows the measurement results for three different concentrations of CRP (10 μg/mL, 1 μg/mL, and 100 ng/mL) using the OCB. For CRP detection, we followed the same fabrication and functionalization processes used for the streptavidin detection. To functionalize the sensing area with the CRP antibody, we first introduced 30 μL of streptavidin with a concentration of 100 μg/mL to the microfluidic channel and incubated for at least 30 min so that the streptavidin molecules were immobilized on the biotin on the sensing area. After rinsing the channel with DI water to remove unbound streptavidin molecules, 30 μL of biotin-conjugated CRP antibody with a concentration of 100 μg/mL was introduced and incubated for at least 30 min so that the biotin part of it was attached to the streptavidin-coated surface, while the CRP antibody covered the surface. The microfluidic channel was rinsed with DI water to remove unbound CRP antibody molecules and filled with it for fine-tuning the optical cavity through polymer swelling. When the optical cavity was ready for the experiments, we introduced 15 μL of CRP protein spiked in tris-HCl buffer with a flow rate of about 0.9 μL/min for about 17 min. Finally, the microfluidic channel was rinsed with DI water, and the average differential value changes were determined by averaging differential values between 8 and 10 min after DI water was introduced. The measured average differential value changes were 0.141 (10 µg/mL), 0.061 (1 µg/mL), and 0.018 (100 ng/mL). Based on the measured average standard deviation in the baseline data with DI water (σ = 0.00284), the LOD for CRP detection is determined to be 43.3 ng/mL (377 pM).

## 4. Discussion and Future Work

The LOD of our OCB for streptavidin detection (1.35 nM) can be improved further. First, the sensing area (2.5 mm^2^) where streptavidin was allowed to be attached was larger than the area that was used for data processing (0.02 mm^2^; the area of a 160 μm diameter circle). If we properly functionalize only this area with sulfo-NHS-biotin, so that streptavidin molecules can be attached within the area of 0.02 mm^2^, then the LOD of the OCB becomes 10.9 pM, assuming streptavidin molecules are attached on the sensing area uniformly. Second, as we discussed earlier, the current functionalization and passivation processes need to be improved further to allow more streptavidin molecules to be attached only at the sensing area. If we consider the sensing area of 0.02 mm^2^ and lose only about 20% of the target molecules (equivalently, 15 µL of 1 µg/mL streptavidin forms a monolayer on 2.5 mm^2^), then we can estimate that the LOD can be improved further by an order of magnitude, to 1.09 pM. Finally, the LOD will be improved proportionally by the amount of sample fluid. This is simply because, to be able to detect the differential value changes, we need a certain number of target biomolecules on the sensing area regardless of the total volume of sample fluid. For a lower concentration sample, the sample fluid with a larger volume will contain enough biomolecules to cause the differential value to change to greater than 3σ. For example, the LOD can go down to 109 fM, which is a 10-times smaller concentration than the 1.09 pM given by the previous LOD estimation, with a sample volume of 150 μL (i.e., 10 times the 15 μL volume used in the previous analysis). The same analysis can be applied to the LOD for the CRP detection. For the smaller sensing area of 0.02 mm^2^, the LOD for CRP can be improved to 3.02 pM from 377 pM with the sensing area of 2.5 mm^2^. With improved functionalization and passivation processes, assuming only 20% of target molecules will be lost, the LOD for CRP will be improved further to 302 fM. Finally, with the sample volume of 150 µL, the LOD can reach up to 30.2 fM.

The design presented in the work was optimized for a silver thickness of 20 nm. It is possible to design an optical cavity structure with a better LOD with a thicker silver layer. A thicker silver layer will increase the reflectance of the partially reflective mirrors which will, in turn, increase the quality (Q) factor of the optical cavity (i.e., a sharper resonance curve). With a sharp resonant response, the intensity changes of two wavelengths will become steeper, as the sensing layer’s thickness increases, than those with the current design. This will enhance the differential value change and, therefore, improve the LOD. However, a thicker silver layer will also increase the absorption of light, increasing the optical loss of two laser diodes. The optical loss at each partially reflective mirror will reduce the sharpness of the resonant response. Therefore, an improved optical cavity design with a thicker silver is possible, but it has to be experimentally optimized due to these conflicting phenomena.

## 5. Conclusions

The optical cavity-based biosensor (OCB) has been developed for the purpose of POC biosensing. It is a label-free system detecting the local refractive index change due to the adsorption of target biomolecules on the receptor molecules. It is a low-cost system with simple and straightforward fabrication processes and low-cost parts and components, which achieves high sensitivity by employing the differential detection method. To demonstrate the limit of detection (LOD) of the OCB experimentally, we conducted streptavidin and CRP detection tests. For a silver thickness of 20 nm, the optimized optical cavity structure has a cavity height of 8 μm and a SOG thickness of 400 nm for the wavelengths of 830 nm and 904 nm. The fabricated devices have typical layer thicknesses of 22 nm (silver), 410 nm (SOG), 6.4 μm (SU8), and 1.08 μm (UV glue). The SOG surface was functionalized by the vapor-phase deposition of APTES followed by sulfo-NHS-biotin covalent bonding for more reproducible and stable test results. The polymer swelling property was used to fine-tune the optical cavity width. From the triplicate test results for streptavidin detection, the LOD of the OCB was determined to be 1.35 nM with four different concentrations of streptavidin. Human CRP was chosen to demonstrate our OCB’s ability to detect an actual biomarker for the first time. With biotin-conjugated CRP antibodies as the receptor molecules, the OCB successfully detected CRP with an LOD of 377 pM. All measurements were done using a small sample volume of 15 µL in a short time, less than 30 min, once the optical cavity reached the measurable condition after the fine-tuning process. We showed that the LOD of our OCB can be improved further into the femto-molar range for both streptavidin and CRP by reducing the sensing area, improving the functionalization and passivation processes, and increasing the sample volume. The LOD of the OCB could be possibly improved with a thicker silver layer, but it must be experimentally optimized.

## Data Availability

The data presented in this study are available on request from the corresponding author.
